# Association of Marek’s Disease induced immunosuppression with activation of a novel regulatory T cells in chickens

**DOI:** 10.1371/journal.ppat.1006745

**Published:** 2017-12-21

**Authors:** Angila Gurung, Nitin Kamble, Benedikt B. Kaufer, Ansar Pathan, Shahriar Behboudi

**Affiliations:** 1 The Pirbright Institute, Ash Road, Woking, United Kingdom; 2 College of Health and Life Sciences, Department of Life Sciences, Brunel University, London, United Kingdom; 3 Institut für Virologie, Freie Universität Berlin, Robert-von-Ostertag-Straße, Berlin, Germany; 4 Department of Pathology and Infectious Disease, School of Veterinary Medicine, University of Surrey, Guildford, United Kingdom; USDA-ARS, UNITED STATES

## Abstract

Marek’s Disease Virus (MDV) is an *alphaherpesvirus* that infects chickens, transforms CD4^+^ T cells and causes deadly lymphomas. In addition, MDV induces immunosuppression early during infection by inducing cell death of the infected lymphocytes, and potentially due to activation of regulatory T (Treg)-cells. Furthermore, immunosuppression also occurs during the transformation phase of the disease; however, it is still unknown how the disease can suppress immune response prior or after lymphoma formation. Here, we demonstrated that chicken TGF-beta^+^ Treg cells are found in different lymphoid tissues, with the highest levels found in the gut-associated lymphoid tissue (cecal tonsil: CT), fostering an immune-privileged microenvironment exerted by TGF-beta. Surprisingly, significantly higher frequencies of TGF-beta^+^ Treg cells are found in the spleens of MDV-susceptible chicken lines compared to the resistant line, suggesting an association between TGF-beta^+^ Treg cells and host susceptibility to lymphoma formation. Experimental infection with a virulent MDV elevated the levels of TGF-beta^+^ Treg cells in the lungs as early as 4 days post infection, and during the transformation phase of the disease in the spleens. In contrast to TGF-beta^+^ Treg cells, the levels of CD4^+^CD25^+^ T cells remained unchanged during the infection and transformation phase of the disease. Furthermore, our results demonstrate that the induction of TGF-beta^+^ Treg cells is associated with pathogenesis of the disease, as the vaccine strain of MDV did not induce TGF-beta^+^ Treg cells. Similar to human haematopoietic malignant cells, MDV-induced lymphoma cells expressed high levels of TGF-beta but very low levels of TGF-beta receptor I and II genes. The results confirm that COX-2/ PGE2 pathway is involved in immunosuppression induced by MDV-lymphoma cells. Taken together, our results revealed a novel TGF-beta^+^ Treg subset in chickens that is activated during MDV infection and tumour formation.

## Author summary

Treg cells are crucial for the maintenance of tolerance and control of immune responses, especially during viral infection and tumour formation. Marek’s Disease Virus (MDV) infection causes immunosuppression and induces transformation of CD4^+^ T cells in chicken. Here we demonstrate that a population of chicken CD4^+^ T cells express inhibitory molecules including TGF-beta and have immune-regulatory properties. TGF-beta^+^ Treg cells are detected in different chicken lymphoid tissues, the highest being detected in cecal tonsils. Chicken lines susceptible to MDV-induced lymphoma formation have higher frequencies of TGF-beta^+^ Treg cells compared to the MDV resistant chicken line. Infection of chicken with a virulent MDV increased the numbers of TGF-beta^+^ Treg cells, which was not changed after infection with the MDV vaccine strain. MDV-transformed CD4^+^ T cells produce high levels of TGF-beta, while they express very low levels of TGF-beta receptors compared to non-transformed CD4^+^ T cells. In addition, MDV-induced lymphoma cells express soluble suppressive factors that can inhibit T cell function; however these soluble factors cannot suppress proliferation of the lymphoma cells. For the first time, we identified chicken TGF-beta^+^ Treg cells and demonstrate that these cells are involved in pathogenesis and immunosuppression of MDV infection.

## Introduction

Regulatory T cells (Tregs) are critical for maintenance of immune-homeostasis and immunological tolerance by enforcing negative regulation on T helper (Th) cells. Transcription factor Foxp3 (Foxp3) is a lineage specific factor for human and murine CD4^+^CD25^+^ Treg cells and is crucial for Treg development and function. TGF-beta can bind to the surface of human Foxp3^+^ Treg cells via GARP (LRRC32) a membrane anchoring molecule, and these cells can be classified as activated Treg cells with a highly potent immune-regulatory properties [1–3].

In chickens, CD4^+^CD25^+^ T cells have been classified as Treg cells which are present in most tissues including thymus [4] thus, they are thought to be equivalent to mammalian natural regulatory T cells (nTreg cells). Interestingly, expression of Foxp3 is restricted to jawed vertebrate and no Foxp3-like genes has been identified in the chicken genome [5]. Therefore, CD25 is currently the only marker for identification of Treg but CD25 is also a marker for activated T cells [2]. Apart from immune regulatory activity, Tregs cells are implicated in progression of the tumour and pathogenesis of viral as well as bacterial diseases in humans and mice. Depletion of tumour-induced Treg cells can reduce tumour progression via the activation of T cell responses [6–9]. It has been postulated that Treg cells are involved in the development of malignant lymphomas, however, their role is more complex in lymphoma than that in other cancers such as carcinomas [10]. Transformed CD4^+^ T cells (lymphoma) may express inhibitory markers, and suppress anti-tumour immunity. Therefore, Treg cells isolated from malignant lymphoma patients can be categorized as primary Treg cells or malignant Treg cells [10].

Marek’s Disease (MD) is caused by a highly contagious *alphaherpesvirus*, Marek’s Disease Virus (MDV), leading to development of malignant CD4^+^ T cell lymphoma in domestic chickens. The pathogenesis of the MD can be classified into three distinct phases: i) the early cytolytic phase with the infection of B and T cells which is associated with transient immunosuppression, ii) the latency phase which is defined by absence of viral protein expression and viral replication and iii) the transformation phase that leads to a deadly lymphoma. In MD-susceptible chicken lines, a second cytolytic phase may occur, resulting in atrophy of lymphoid tissues and a severe immunosuppression [11]. During the early phase of infection, both T and B cells are the predominant target cells for MDV infection in the thymus, spleens and the bursa of Fabricius. MDV replicates in the infected B and T cells, resulting in a depletion of lymphocytes and transient immunosuppression in the host [12]. The activation of T cells via the antigen presenting B cells is important for the establishment of primary infection, as the resting T cells are resistant to the MDV infection [13].

Here we identified and characterized TGF-beta^+^ Treg cells in chickens and demonstrated that MD-susceptible chicken lines have higher levels of TGF-beta^+^ Treg cells. Infection with virulent virus, but not vaccine strains, increased the number of TGF-beta^+^ Treg cells. Interestingly, MDV-induced lymphoma cells adopt a Treg phenotype and release soluble molecules that can inhibit T cell function. Taken together, our results suggest that activated Treg cells may be involved in MDV-induced immunosuppression in chickens.

## Results

### A population of CD4^+^ T cells express membrane bound TGF-beta

**Fig 1:**
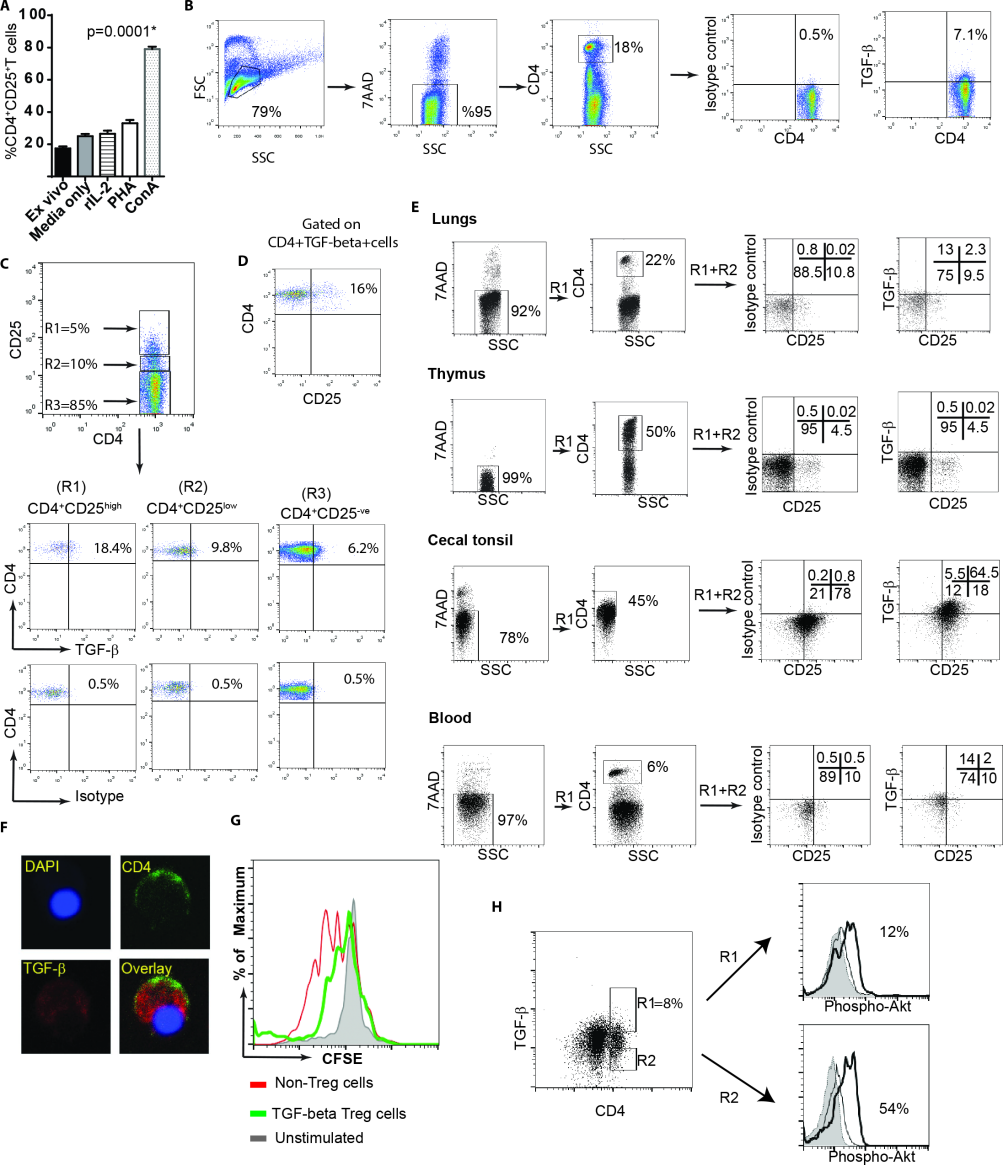
Identification and characterization of chicken TGF-beta^+^ Treg cells. A) Mononuclear cells isolated from the spleens of 3 weeks old RIR birds were independently cultured in the presence of media only, rIL-2, PHA or Con-A at 40°C. After 72 hrs, the cells were stained with anti-CD4, and anti-CD25; 7AAD staining was used for dead cell exclusion. Data (mean ± SD) represents the percentages of CD4^+^CD25^+^ T cells in different cell culture conditions. The results are representative of three independent experiments with three biological replicates. (B) Representative FACS profiles and percentages TGF-beta^+^CD4^+^ T cells within CD4^+^ T cell population are shown. Mononuclear cells, isolated from spleens of 3 weeks-old naïve RIR chickens, were stained with anti-CD4-PE, anti-TGF-beta-APC mAbs and/ or isotype controls. The 7AAD was added for exclusion of dead cells. C) Representative of FACS profile and percentages TGF-beta^+^CD4^+^ T cells within three subpopulations: CD25^high^ (R1); CD25^low^ (R2); CD25^-ve^(R3) CD4^+^ T cells are shown. D) The percentages of CD25^+^ cells within TGF-beta^+^CD4^+^ T cells are shown. E) TGF-beta expressions on gated CD4^+^ T cells isolated from lungs, thymus, cecal tonsil, and blood of 3-weeks old RIR chickens were analysed using flow cytometry. The number in each quadrant represents percentages of the cells. The results are representative of data obtained from one out of six chickens. F) The intracellular staining of TGF-beta (Red) and cell surface CD4 (green) are shown using confocal microscopy. Mononuclear cells from spleen were stained with primary mouse anti-chicken CD4 (IgG2b), and mouse anti-TGF-beta (IgG1) monoclonal antibodies followed by staining with anti-mouse IgG1-Alexa Flur 488 or IgG2b-Alexa Flur 568 secondary antibodies, respectively. DAPI was used to visualize the nucleus. G) *In vitro* inhibitory function of TGF-beta^+^CD4^+^ T cells using a CFSE based proliferative assay 72 hrs after co-culture. CFSE-labelled responder cells were co-cultured with or without TGF-beta^+^CD4^+^ T cells in the presence or absence of 2.5 μg/mL Con-A. Filled grey profiles represent non-stimulated controls without co-culturing with TGF-beta^+^CD4^+^ T cells. Empty profiles represent stimulation with 2.5 μg/mL Con-A in co-culture with TGF-beta^+^ CD4^+^ T cells (green lines), or without co-culturing with TGF-β^+^CD4^+^ T cells (red lines). H) *Ex vivo* splenocytes isolated from RIR chickens were stimulated with PMA and stained for CD4 and TGF-beta. The levels of Phospho-AKT were analysed using Phosflow by gating on CD4^+^TGF-beta^+^ (R1) or CD4^+^TGF-beta^-^ (R2) T cells. Filled grey profiles represent isotype control, thin lines represent non-activated cells, and thicker lines represent the expression of Phospho-AKT in PMA-activated cells. The percentages of cells expressing Phospho-AKT for activated cells are shown for the two sub-populations. The results represent of at least three independent experiments.

CD25 molecule is not only a marker for identification of human and murine Treg cells but also for activated T cells. We confirmed this observation in chickens by analysing the surface expression levels of CD25 on CD4^+^ T cells isolated from spleens (3 weeks old Rhode Island Red (RIR) chickens) upon activation with Con-A or PHA. As expected, addition of Con-A or PHA to splenocytes increased the expression of CD25 molecules on the activated CD4^+^ T cells 3 days after *in vitro* activation (Fig 1A). Another marker for identification of a subpopulation of human Treg cells is membrane bound TGF-beta. To examine whether chicken Treg cells also express membrane bound TGF-beta, mononuclear cells from different tissues (blood, spleen, lungs, cecal tonsil, and thymus) were isolated from RIR chickens (3 weeks old) and stained with anti-CD4 and anti-TGF-beta mAbs. Flow cytometry analysis of these cells revealed that a subpopulation of chicken CD4^+^ T cells express membrane bound TGF-beta (Fig 1B). The next step was to determine whether membrane bound TGF-beta are preferentially expressed on CD4^+^CD25^+^ T cells or CD4^+^CD25^-^ T cells. CD4^+^ T cells were subdivided into three subpopulations; CD25^-^, CD25^low^ and CD25^high^ CD4^+^ T cells, and the expression of TGF-beta was analysed in these three subpopulations. The data revealed that TGF-beta is expressed on both CD4^+^CD25^-^ and CD4^+^CD25^+^ T cells, however a higher percentages of CD4^+^CD25^high^ T cells expressed TGF-beta compared to CD4^+^CD25^-^ T cells (Fig 1C). Only 15–20% of TGF-beta^+^CD4^+^ T cells expressed CD25 molecules (Fig 1D), suggesting that TGF-beta^+^CD4^+^ T cells and CD4^+^CD25^+^ T cells are two distinct cell types with some overlap. Flow cytometry analysis of surface expression of TGF-beta on CD4^+^ cells show that TGF-beta^+^CD4^+^ T cells are present in various lymphoid tissues including spleen, cecal tonsils, lungs, and blood of chickens (Fig 1B, C and 1E). Intriguingly, unlike CD4^+^CD25^+^ T cells, TGF-beta^+^CD4^+^ T cells were not found in thymus of chickens (Fig 1E), indicating that TGF-beta^+^CD4^+^ T cells are induced in the periphery. In addition, confocal microscopy confirmed that a population of primary CD4^+^ T cells express TGF-beta as both an intracellular and membrane bound form (Fig 1F).

One of the immune-regulatory functions of human and murine Tregs is their ability to inhibit T cell proliferation *in vitro*. Hence, we utilized a typical inhibitory T cell proliferation assay to examine the inhibitory properties of chicken TGF-beta^+^CD4^+^ T cells. The cells were isolated from spleens of 3-weeks old RIR chickens and the CFSE-labelled responder cells were co-cultured with TGF-beta^+^CD4^+^ T cells or TGF-beta^-^CD4^+^ T cells and were stimulated with Concanavalin (Con)-A, and the proliferation of T cells were analysed 3 days after co-culture using flow cytometry. The result demonstrated that TGF-beta^+^CD4^+^ T cells reduced T cell proliferation *in vitro* (Fig 1G), suggesting that these cells exhibit immune-regulatory activity.

A defining characteristic of human CD4^+^ Treg cells is their defect in phosphorylation of AKT at S473 [14]. To determine the AKT phosphorylation levels in chicken TGF-beta^+^ Treg cells, mononuclear cells from 3-weeks old RIR chickens were isolated and were stimulated with PMA for 15 minutes. AKT phosphorylation at S473 were analysed within TGF-beta^+^ Treg cells and TGF-beta^-^CD4^+^ T cells using Phosflow. Fig 1H demonstrates that chicken TGF-beta^+^ Treg cells (R1 gate) have lower AKT activation at S473 compared to TGF-beta^-^ CD4^+^ T cells (R2 gate), confirming that chicken TGF-beta^+^ Tregs have also a defect in phosphorylation of AKT.

**Fig 2:**
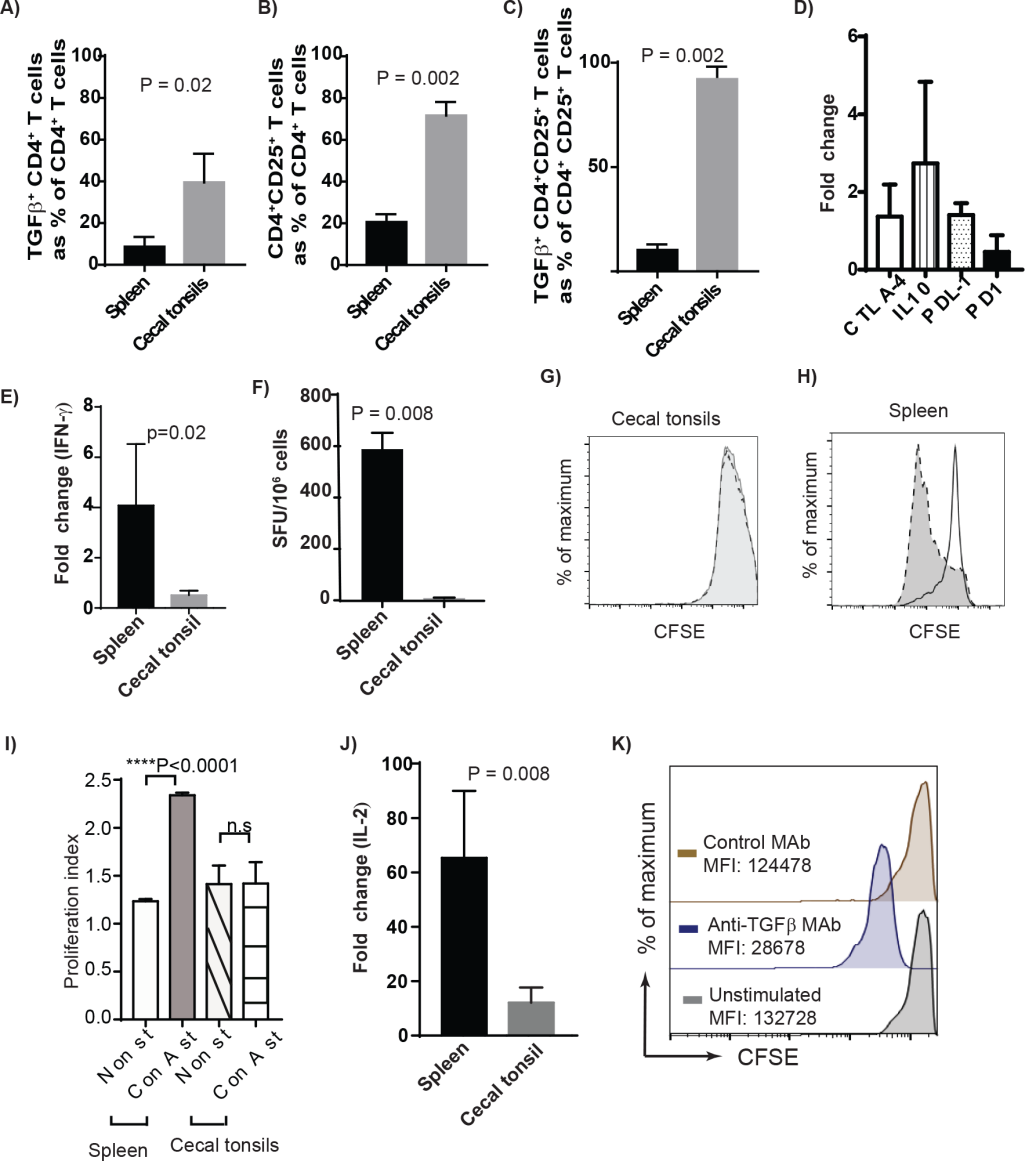
High concentrations of Treg cells in cecal tonsils is associated with immune-privileged microenvironment. Mononuclear cells were isolated from spleens or cecal tonsils of six 3-weeks old RIR birds and were stained with anti-CD4-PE, CD25-FITC, and TGF-beta-APC or isotype controls. 7AAD was used for dead cell exclusion, and the cells were analysed using FACS. Percentages of **(A)** TGF-beta^+^CD4^+^ T cells (**B),** CD4^+^CD25^+^ T cells **(C)** TGF-beta^+^CD4^+^CD25^+^ T cells in the spleens and cecal tonsils are shown. **(D)** Relative quantification of the CTLA-4, IL-10, PDL1 and PD1 molecules in cecal tonsils over spleen mononuclear cells. Fold change was calculated in non-stimulated cecal tonsils considering normalized Ct value for respective molecule from spleen as baseline. **(E)** Relative quantification of fold change in IFN-γ gene in cecal tonsils and spleens after 4 h of stimulation with PMA/Ionomycin were determined using RT-PCR. **(F)** The frequencies of IFN-γ producing cells in cecal tonsils and spleens were determined using chicken-IFN-gamma ELISPOT assay 18 hrs after PMA/Ionomycin stimulation. (**G)** CFSE histograms from one representative of T cell proliferation in cecal tonsils and (**H)** in splenocytes are shown following stimulation with 2.5 μg/ml Con-A. Empty profiles with solid line represent non-stimulated control cells and the grey shaded area represents Con A-stimulated cells. (**I)** Graphical representation of proliferation index in Con-A stimulated and non-stimulated mononuclear cells from spleens and cecal tonsils of seven birds are shown. **(J)** Relative quantification of the IL-2 cytokine gene in PMA/Ionomycin stimulated cecal tonsils and spleen mononuclear cells. The fold change in mRNA for IL-2 cytokine in stimulated cells was calculated over non-stimulated cells after normalizing against housekeeping gene. **(K)** anti-TGF-beta blocking antibody partially restored the proliferation of mononuclear cells isolated from CT using CFSE-based proliferation assay. Data are shown as means with standard deviations (error bars) of at least of three independent experiments. ns, not significant.

As described above, we demonstrated that TGF-beta^+^ Treg cells are present in various tissues of chickens. However, the highest frequencies of these cells were found in cecal tonsils (CT) as detected using flow cytometry (Fig 1E). A representative of Forward and Sideward scatter of cells isolated from different tissues including spleens, blood, lungs, thymus and CT are shown (S1A Fig). Exclusion of dead cells (7AAD positive cells) (Fig 1E) and the light microscopy of the cells isolated from CT demonstrated that these cells are alive and intact (S1B Fig). Various subsets of Treg cells that promote immunological non-responsiveness have been suggested to be involved in sustaining immune privilege in the gastrointestinal tract under normal homeostatic conditions. Here we demonstrated that the frequencies of both TGF-beta^+^CD4^+^ cells and CD4^+^CD25^+^ T cells in the CT of RIR chickens (3 weeks old) were significantly higher than that observed in the spleens (p = 0.002, n = 8) (Fig 2A, 2B and 2C). We also examined the differential expression levels of inhibitory molecules including CTLA-4, IL-10, PDL1 and PD1 in CD4^+^ T cells from CT and spleens of RIR chickens using semi-quantitative RT-PCR. Chicken CD4^+^ T cells isolated from CT expressed significantly higher levels of inhibitory genes including CTLA-4, PD1 and IL-10 compared to the cells isolated from the spleens (Fig 2D). Another characteristic of Treg cells is their ability to inhibit the production of proinflammatory Th1 cytokines such as IFN-gamma. To analyse the differential IFN-gamma expression in CT and spleens, mononuclear cells isolated from spleens and CT of RIR chickens were stimulated with PMA and Ionomycin for 4hrs and the expression of IFN-gamma gene was determined using semi-quantitative RT-PCR. The results confirmed that the cells isolated from CT are unable to up-regulate the expression of IFN-gamma gene (Fig 2E). Similarly, we observed a significantly lower frequencies of IFN-gamma producing T cells in mononuclear cells isolated from CT, stimulated with PMA and Ionomycin for 18 hrs, compared to that from the spleens using chicken IFN-gamma ELISPOT assay (Fig 2F). Generally, Treg cells exhibit reduced proliferative response in the absence of rIL-2 *in vitro*. The unresponsiveness of mononuclear cells isolated from CT was demonstrated in a CFSE-based proliferation assay. In contrast to mononuclear cells from spleens, the cells isolated from CT did not proliferate in response to Con-A stimulation (Fig 2G and 2H). Fig 2I shows proliferation index of mononuclear cells from CT cells compared to splenocytes. The results show that CT cells are unresponsive and do not proliferate after the stimulation. One of the mechanisms involved in Treg-induced suppression is their ability to inhibit IL-2 mRNA expression [15]. To examine whether T cells from CT can respond to the stimulation and up-regulate IL-2 genes, CD4^+^ T cells from CT and spleens of RIR chickens were stimulated with PMA and Ionomycin for 4 hrs and IL-2 mRNA expression was analysed using RT-PCR. IL-2 expression was not upregulated in CT, while IL-2 gene expression was significantly increased in the cells isolated from spleens (Fig 2J). To examine the role of TGF-beta in providing an inhibitory microenvironment in CT, the mononuclear cells from CT were treated with either anti-TGF-beta or control MAbs and the cells were stimulated with Con A, and the proliferation was analysed using a CFSE-based proliferation assay. The treatment of mononuclear cells isolated from CT with anti-TGF-beta blocking antibody, but not an isotype control MAb, increased their ability to proliferate *in vitro* (Fig 2K). Con-A was used in all T cell proliferation assays performed in this study. Unlike Con-A, PMA (ranging from 12.5–100 ng/ml) did not stimulate chicken T cell proliferation (S2 Fig). Taken together, the above results suggest that high concentration of TGF-beta^+^ Treg cells in CT is associated with immune-privileged microenvironment in the largest gut associated lymphoid tissues in chickens, and TGF-beta is involved in the induction of unresponsiveness to the stimulation in CT.

**Fig 3:**
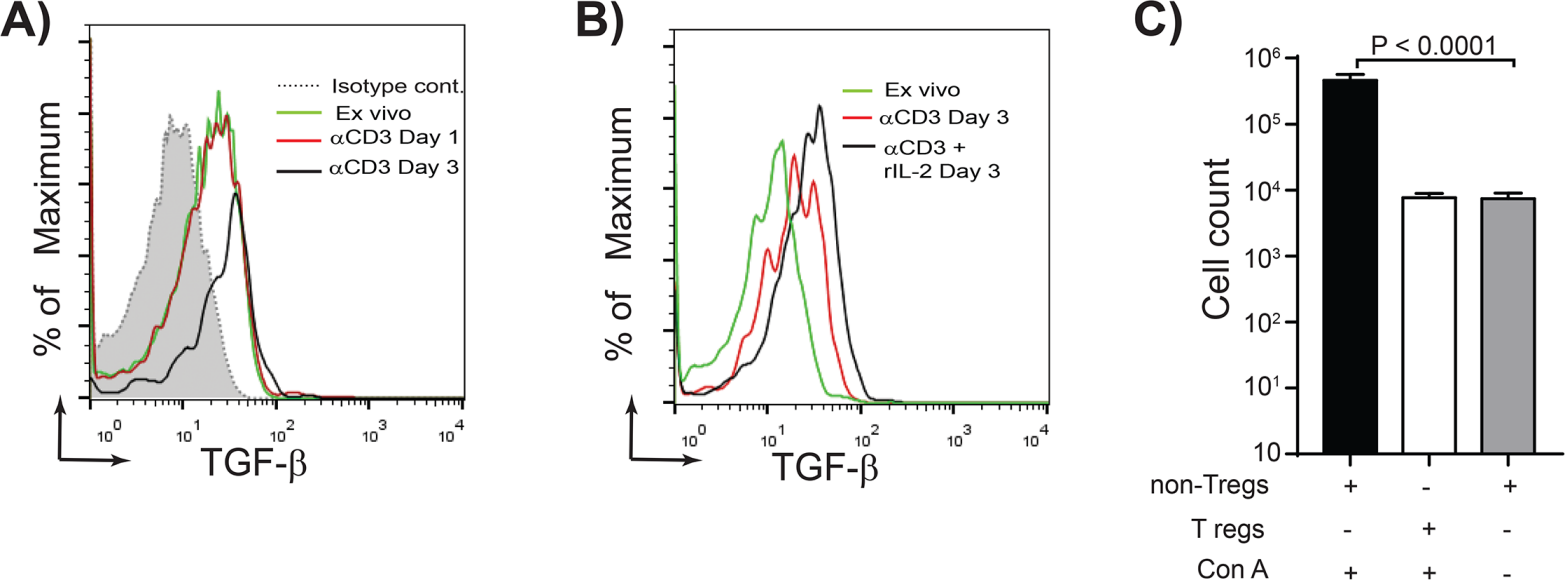
Induction of TGF-beta^+^CD4^+^ T cells in vitro. **(A)** Representative of overlapping histogram demonstrating the expression levels of TGF-beta on gated CD4^+^ T cells isolated from 3-weeks old RIR chickens. *Ex vivo* CD4^+^ T cells (green line), CD4^+^ T cells stimulated with anti-CD3 mAb for 24 hours (red line), and 72 hrs (black line) are shown. Dotted line represents cells stained with isotype control antibody, **(B)** Compares the expression levels of TGF-beta on CD4^+^ T cells *ex vivo* (green line), or cells stimulated with anti-CD3 and rIL-2 (black line), and cells stimulated with only anti-CD3 mAb (red line) at 72 hours after stimulation. **(C)** The numbers of live cells (7AAD negative) are shown after co-cultured of the responder cells with non-Treg cells (TGF-beta^-^ Treg cells) (black bar) or Treg cells (TGF-beta^+^CD4^+^ T cells) (open bar) 72hrs after stimulation with Con-A. Grey bar represents the numbers of live cells in co-culture of the responder cells and TGF-beta^-^CD4^+^ T cells without stimulation. A representative of three independent experiments is shown.

To understand how TGF-beta^+^ Treg cell are generated *in vivo*, we developed a protocol for generation of chicken Treg cells *in vitro*. CD4^+^ T cells isolated from RIR chickens were cultured for 1–3 days in the presence of plate-bound anti-chicken CD3 antibody. TGF-beta expression was up-regulated on CD4^+^ T cells 3 days after culture with anti-CD3 antibody (Fig 3A). Addition of rIL-2 increases the expression of TGF-beta on CD4^+^ T cells (Fig 3B). These *in vitro* generated TGF-beta^+^ Treg cells inhibited T cell proliferation upon Con-A-stimulation (Fig 3C), indicating that these cells have immune-regulatory activity. Activation of CD4^+^ T cells or splenocytes with PMA, PHA or Con-A did not induce TGF-beta expression on chicken CD4^+^ T cells (ranging from 4.5 to 7% of CD4^+^ T cells; p = 0.6).

### Higher frequencies of TGF-beta^+^ Treg cells is associated with susceptibility to MDV-induced CD4^+^ T cell lymphoma

**Fig 4:**
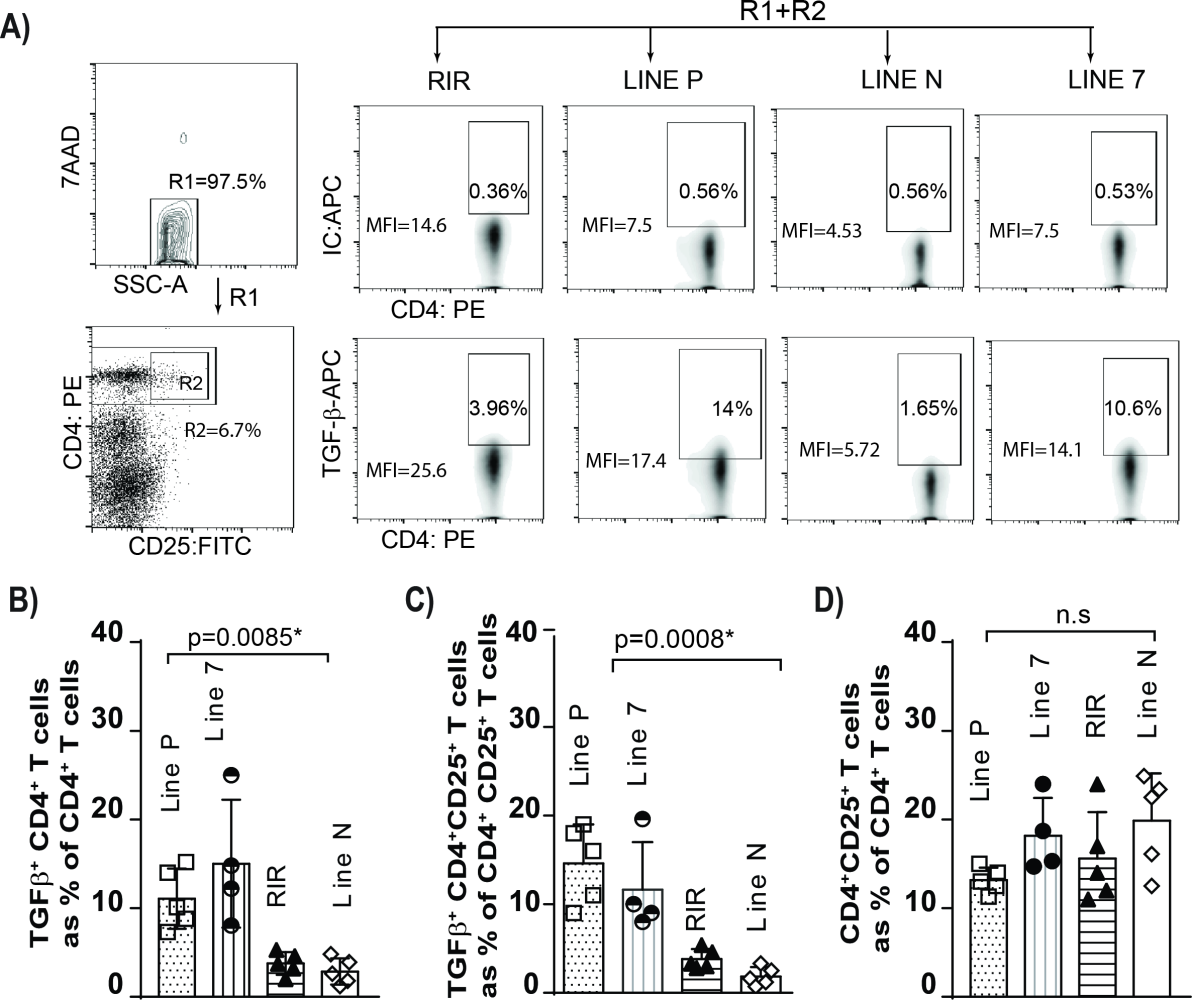
High concentrations of TGF-beta^+^CD4^+^ T cells in susceptible birds to virus-induced lymphoma. **A)** Gating strategy for detection of TGF-beta+ Treg cells are shown. Mononuclear cells were isolated from spleens of 3 weeks-old naïve chicken lines, and were surface stained with anti-CD4-PE, anti-CD25-FITC and anti-TGF-beta-APC mAbs or isotype controls. The 7AAD staining was used for dead cell exclusion. The percentages and mean fluorescent intensity (MFI) of CD4^+^ cells expressing TGF-beta are shown in FACS density plots. The percentages of **(B)** TGF-beta^+^CD4^+^ T cells, **(C)** TGF-beta^+^CD4^+^CD25^+^ T cells and **(D)** CD4^+^CD25^+^ T cells in different chicken lines are shown. The values are representative from five different birds for each group. The mean ± SD value are shown. * indicates a statistically significant difference (*P* < 0.05); ns, not significant.

The genetic background plays an important role in the susceptibility of chicken lines to the formation of MDV-induced lymphoma. While, the resistant lines display fewer tumours than susceptible lines, the resistant lines will develop tumours, just at a much-reduced rate (*e*.*g*. line P and line 7 birds being susceptible and line N birds being resistant to lymphoma formation). MDV infection induces immunosuppression, however the exact mechanisms involved in MDV-induced immunosuppression is still unknown [11]. Mononuclear cells from the spleens of aged matched naïve chickens from four different chicken lines (line P, line 7, line N and RIR) were isolated and counted prior to staining for flow cytometry analysis. The results showed that there was no significant difference in the total numbers of splenocytes (ranging from 52 to 68 x 10^6^ cells) and total CD4^+^ T cells among these chicken lines (p = 0.6). The cells were stained with anti-CD4, anti-CD25 and anti-TGF-beta antibodies and the percentages of TGF-beta^+^CD4^+^ T cells, TGF-beta^+^CD4^+^CD25^+^ and CD4^+^CD25^+^ T cells were analysed using flow cytometry. The MD-susceptible line P and 7 had significantly higher percentages of TGF-beta^+^CD4^+^ T cells (Fig 4B) and TGF-beta^+^CD4^+^CD25^+^ T cells (Fig 4C) than the MD-resistant line N, demonstrating that there is an association between high frequencies of TGF-beta^+^ Treg cells in the spleens and susceptibility to MDV-induced lymphoma formation (p = 0.0008). Intriguingly, there were no significant differences in the frequencies of CD4^+^CD25^+^ T cells (Fig 4D) among these chicken lines.

### Infection with an oncogenic MDV increases the frequencies of TGF-beta^+^ Treg cells in the susceptible line

**Fig 5:**
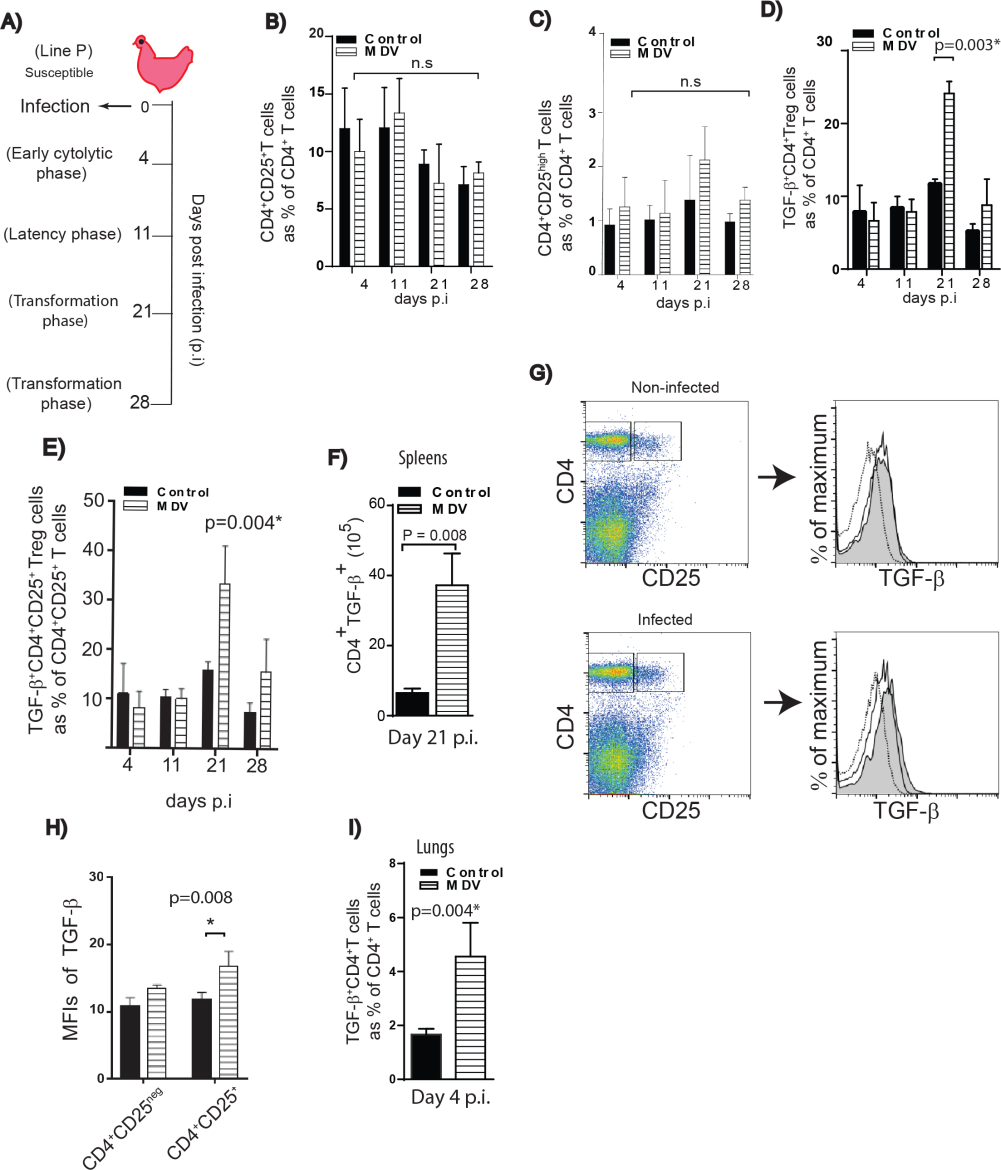
Infection with virulent virus induces TGF-beta^+^ Treg cells in vivo. **(A)** A schematic diagram of animal trials is shown. Day old line P chicks were mock-infected or infected with the virulent virus via intra-tracheal route. Spleens were taken from 5 birds at different time points post infection. Percentages of **(B)** CD4^+^CD25^+^ T cells **(C)** CD4^+^CD25^high^ T cells **(D)** TGF-beta^+^CD4^+^ T cells **(E)** TGF-beta^+^CD4^+^CD25^+^ T cells are shown. **(F)** Absolute numbers of TGF-beta+ Treg cells are shown at 21 dpi. **(G)** Representative flow cytometry plots isolated from splenocytes of non-infected and MDV-infected chickens depicting the expression levels of TGF-beta within CD4^+^CD25^-^ or CD4^+^CD25^+^ T cell at 21 dpi. Dashed lines (Isotype control), solid line (CD4^+^CD25^-^ T cells), and shaded grey (CD4^+^CD25^+^ T cells). **(H)** Bar graph (mean ± SD) shows of MFIs of TGF-beta expression within CD4^+^CD25^+^ T cells and CD4^+^CD25^-^ T cells in non-infected and MDV infected chickens (n = 5). **(I)** The percentages of TGF-beta^+^CD4^+^ T cells within mononuclear cells isolated from the lungs at 4 dpi are depicted. * indicates a statistically significant difference (*P* < 0.05); ns, not significant.

Virulent MDV strains cause immunosuppression and efficiently transform CD4^+^ T cells resulting in deadly lymphoma [11]. To assess the impact of MDV infection on TGF-beta^+^ Treg cells *in vivo*, one day old line P chickens were infected via intra-tracheal route with the virulent MDV strain RB1B. Spleen samples were taken from infected and mock-infected birds during (1) early phase of infection at 4 days post infection (dpi), (2) latent phase at 11 dpi, and (3) the transformation phase of MD at 21 and 28 dpi (Fig 5A). Mononuclear cells were stained with anti-CD4, anti-CD25 and anti-TGF-beta mAbs, and 7AAD was used for the exclusion of dead cells. The percentages and absolute numbers of TGF-beta^+^CD4^+^ T cells were determined by flow cytometry. A significant increase (p<0.05, n = 5) in the percentages of TGF-beta^+^CD4^+^ T cells was observed in the spleens of MDV infected birds at 21 dpi (Fig 5D and 5E), while there was no difference in the percentages of total CD4^+^CD25^+^ T cells (Fig 5B) or CD4^+^CD25^high^ T cells (Fig 5C) between the infected and mock infected birds at any time points post infection. The absolute numbers of TGF-beta^+^ Treg cells were calculated and the data confirm that MDV-infection leads to an increase in both percentages and absolute numbers of TGF-beta^+^ Treg cells at 21 dpi (Fig 5F). However, no difference was found in the percentages or absolute numbers of TGF-beta^+^ Treg cells between infected and non-infected birds in the spleens at 4, 11, or 28 dpi (Fig 5). Analysis of mean fluorescent intensities (MFI) of TGF-beta expression on CD4^+^ T cells demonstrated that TGF-beta expression levels were significantly increased on CD4^+^CD25^+^ T cells (p = 0.008, n = 5), but not on CD4^+^CD25^-^ T cells, at 21 dpi (Fig 5G and 5H).

As MDV initially infects lung epithelial cells, we hypothesized that TGF-beta^+^ Treg cells may be induced in the lungs prior to an increase in their frequencies in spleens. FACS analyses revealed that a higher percentage of TGF-beta^+^ Treg cells are detected in the lung tissues isolated from MDV-infected birds compared to mock-infected chickens at 4 dpi (p = 0.004) (Fig 5I), while no increase in the percentages of these cells were observed in the spleens at this time. There was also no difference in the percentages of CD4^+^CD25^+^ (ranging from 10.5 to 11.5% of CD4^+^ T cells; p = 0.4) or CD4^+^CD25^high^ (ranging from 4.5 to 5.3% of CD4^+^ T cells; p = 0.4) T cells in infected and non-infected lung tissues. The results indicate that the induction of TGF-beta^+^ Treg cells occur at an earlier stage of infection in the lungs.

### An increase in the levels of TGF-beta^+^ Treg cells is associated with pathogenesis of the disease

**Fig 6:**
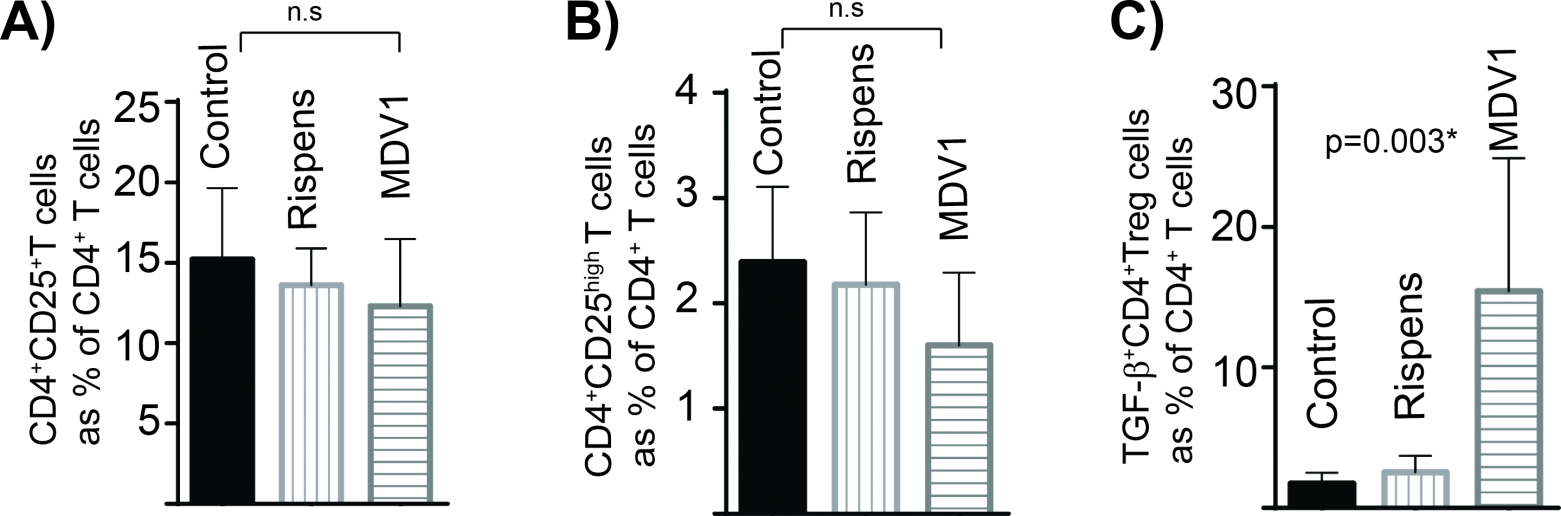
Induction of TGF-beta^+^ Treg cells is association with pathogenesis of the disease. Day-old RIR chicks were administered via intra-abdominal route with 1,000 PFU cell associated RB1B (n = 14), 1,000 PFU cell associated CVI988-Rispens (n = 8), or CEF only (n = 8). The spleens were taken at 21 dpi and the percentages of TGF-beta^+^ Treg cells were determined using FACS. Data are mean ± SD **(A)** CD4^+^CD25^+^ T cells **(B)** CD4^+^CD25^high^ T cells, and **(C)** TGF-β^+^CD4^+^ Treg cells. * indicates a statistically significant difference (*P* < 0.05); ns, not significant.

In this animal experiment, we set to address three questions regarding to the effects of MDV infection on chicken TGF-beta^+^ Treg cells; Does the expansion occur (i) in other chicken lines (ii) upon infection via other routes (iii) with oncogenic and vaccine strains of the virus. To address these questions, RIR birds, an outbred chicken line which is moderately susceptible to MDV lymphoma formation, were selected for this experiment. The birds were infected via the intra-abdominal route with the oncogenic virus (RB-1B), the vaccine strains (CVI988-Rispens) or non-infected CEF cells (mock infected group). This route of infection is routinely used to study MDV pathogenesis. Similar to line P birds, there were no changes in the frequencies of total CD4^+^CD25^+^ T cells (Fig 6A), or CD4^+^CD25^high^ T cells (Fig 6B) on 21 dpi. In contrast, the percentages (Fig 6C) of TGF-beta^+^CD4^+^ T cells were increased (p = 0.003) in the MDV-infected birds. The attenuated MDV vaccine CVI988-Rispens, (98% sequence identity with RB1B) which can infect and replicate in chickens, but cannot induce lymphoma formation, did not induce TGF-beta^+^ Treg (Fig 6C). We also assessed the absolute numbers of TGF-beta Treg cells in the vaccinated group and compared it with that in the control birds. In average, the numbers of splenocytes isolated from vaccinated group were not significantly increased (60–87 x 10^6^ cells per spleen) compared to the CEF control group (61–83 x 10^6^ cells per spleen) at 21 dpi. Similarly the absolute numbers of TGF-beta Treg cells were not altered after vaccination (7.3± 1.2 x 10^5^ cells in vaccinated compared to 7.62 ± 0.85 10^5^ cells in the control group per spleen).These results reveal that the induction of TGF-beta^+^ Treg cells is associated with pathogenesis of the disease and it can also occur in outbred chickens.

Next we examined if the oncogenic RB1B strain also induces TGF-beta Treg cells in a MDV resistant chicken. One day old line N chickens were infected with 1,000 pfu RB1B via intra-abdominal route and the frequencies of TGF-beta Treg cells were compared with the control group (non-infected) 3-weeks post infection using flow cytometry as described above. As expected, no obvious gross pathology was observed in the infected MDV resistant chickens at 3-weeks post infection. The results demonstrated that the infection with the oncogenic virus does not increase the frequencies of TGF-beta Treg cells in the MDV resistant chicken line (TGF-beta Treg cells as % of CD4 T cells were 1.95± 0.54 in the infected chickens compared to %1.86± 0.47 in the control group).

### TGF-beta^+^ MDV-transformed lymphoma cells exert immuno-regulatory activity

**Fig 7:**
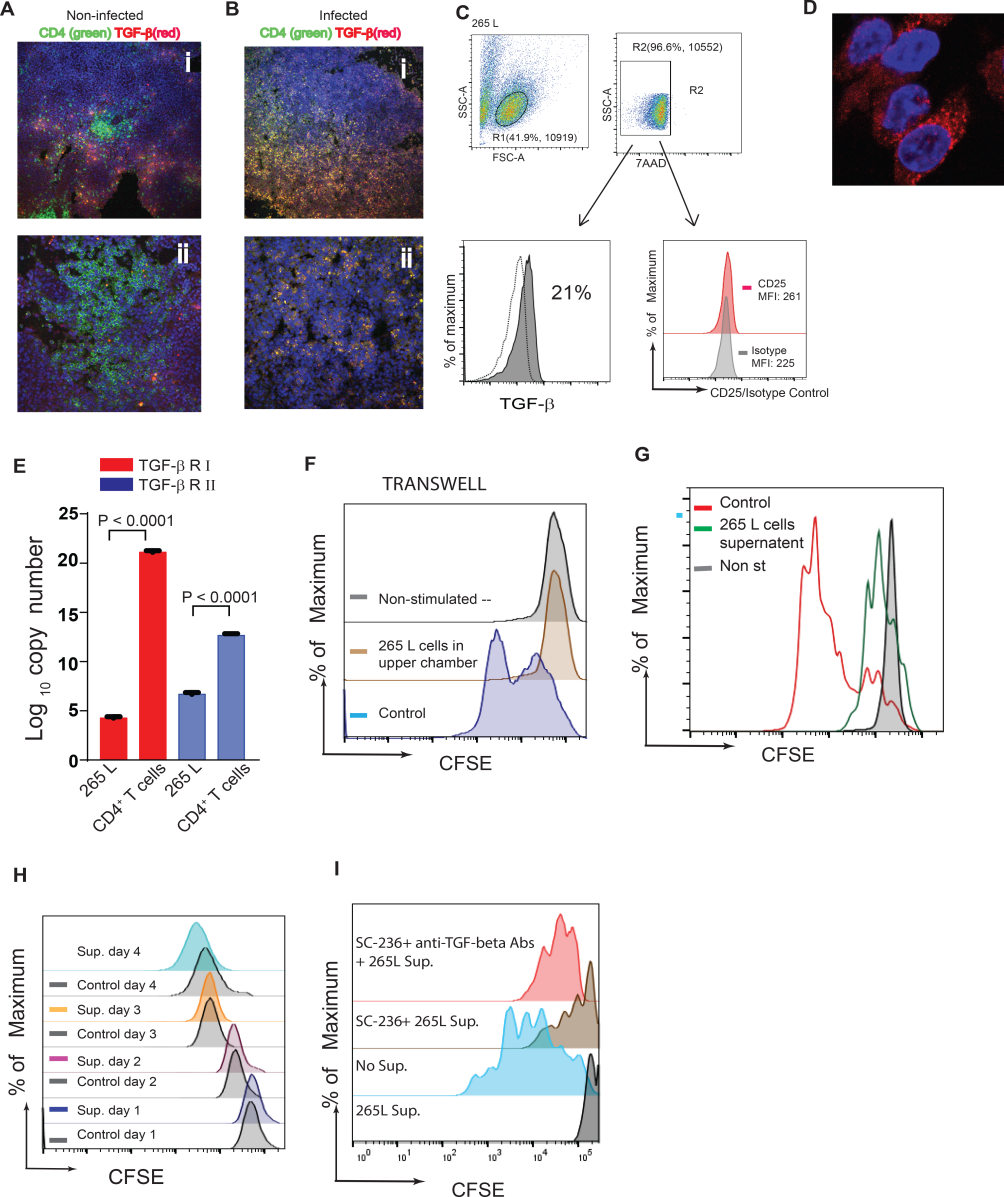
MDV-induced CD4^+^ lymphoma cells express TGF-beta. Confocal microscopy of spleen sections from non-infected (**Ai** and **Aii**; two different magnifications) and MDV-infected birds (**Bi** and **Bii**; two different magnifications) are shown. CD4^+^ T cells (green) and TGF-beta^+^ cells (red) and nuclei (DAPI; Blue) are depicted. **(C)** A representative FACS histogram showing membrane bound TGF-beta on MDV-induced CD4^+^ lymphoma (265L); dotted lines (isotype control MAb) and shaded grey (anti-TGF-beta mAb). **(D)** Confocal microscopy of MDV-induced CD4^+^ T cell lymphoma cell line (265L) expressing intracellular TGF-beta (red) and nuclei (DAPI; Blue). **(E)** The variation in the absolute copy number of mRNA transcript for TGF-beta receptor I (red) and TGF-beta receptor II (blue) receptor in 265L and primary CD4^+^ T cells. The ct values for TGF-beta receptor I and II were normalized against the GAPDH house keeping gene and plotted in the standard curve. Results are mean ± S.E.M. of five independent replicates. The tissues are representative of six different infected and non-infected birds. The results from tumour cell lines are representative of 10 different experiments. **(F)** 265L lymphoma cells inhibited T cell proliferation in a transwell experiment. Splenocytes (in the lower chamber) were stimulated with Con-A, while 265L lymphoma cells were cultured in the upper chamber. T cell proliferation was analysed 3 days after the stimulation using a CFSE-based proliferation assay. **(G)** Cell culture supernatant from 265L lymphoma cells (5% of total cell culture media) inhibited T cell proliferations. Splenocytes were cultured in 100% media or 95% media and 5% supernatant and T cell proliferation was analysed after Con-A stimulation using CFSE-based proliferation assay. **(H)** 265L lymphoma cells were resistant to the effects of soluble factors. 265L lymphoma cells were cultured in 100% media or 60% supernatant plus 40% media and the proliferation was analysed using a CFSE-based proliferation assay. **(I)**Treatment of the cells with SC-236 (PGE2 inhibitor) alone or in combination with TGF-beta blocking antibody partially restored T cell proliferation.

TGF-beta pathway has been linked to tumour progression because it plays an essential role in modulation of cell proliferation and migration [16]. At different stages of tumour development, TGF-beta can either inhibit or promote tumour growth. There is evidence suggesting that overexpression of TGF-beta is associated with poor prognosis in human cancers [17, 18]. Expression of TGF-beta was analysed in spleen tissues obtained from MDV or mock infected line P chickens 3 months post infection, when gross pathological lesions were evident. Tissues were stained with anti-CD4 and anti-TGF-beta antibodies and analysed by confocal microscopy. In mock infected animals, only a small number of CD4^+^ T cells in the splenic T cell zone co-expressed TGF-beta (Fig 7Ai and 7Aii). In contrast, the majority of CD4^+^ T cells in the infected spleens expressed TGF-beta as demonstrated by co-localization of CD4 and TGF-beta molecules (Fig 7Bi and 7Bii). Similar to the primary lymphoma cells, MDV-transformed CD4^+^ T cell clone (clone 265L; generated from line P) expressed both membrane bound (Fig 7C) and intracellular TGF-beta (Fig 7D), as demonstrated using flow cytometry and confocal microscopy, respectively. Approximately 20% of MDV-induced lymphoma cells expressed membrane bound TGF-beta (Fig 7C), while the majority of the lymphoma cells expressed intracellular TGF-beta (Fig 7D). The expression of TGF-beta was not exclusive to 265L clones, as 15–22% of MSB1 clone also expressed mTGF-beta. Interestingly, MDV-induced lymphoma cells (265L) did not express CD25 molecules (Fig 7C). As TGF-beta can act as both paracrine and autocrine factor to inhibit T cell proliferation, we determined the gene expression levels of TGF-beta receptor I and II in 265 L cells (MDV-induced lymphoma cells from line P) using quantitative RT-PCR. MDV-induce lymphoma cells expressed significantly lower levels of TGF-beta receptors compared to the primary naïve CD4^+^ T cells isolated from line P birds (Fig 7E), suggesting that these cells may be resistant to inhibitory effects of TGF-beta on proliferation. Human lymphoma cells may produce many inhibitory factors such as PGE2, VEGF, IL-10 and TGF-beta that can suppress T cell function, and several of these factors may be involved in the induction of Treg cells. In transwell experiments (splenocytes isolated from RIR birds were cultured in the lower chamber and 265L cells in the upper chamber), 265L cells inhibited Con-A induced T cell proliferation *in vitro* in a contact independent manner (Fig 7F). As expected, addition of cell culture supernatant from 265 L lymphoma cells (as low as 5% supernatant/ cell culture media ratio) also inhibited T cell proliferation 3 days after stimulation (Fig 7G), while the addition of supernatant to the cell culture had no effect on proliferation of lymphoma cells even at high ratios (60% supernatant/ cell culture media ratio). Fig 7I depicts the proliferation rate of lymphoma cells, in the presence (coloured histograms) or absence (grey histograms) of the supernatant, 24–96 hrs after culture using a CFSE-based proliferation assay. The results suggest that MDV-lymphoma cells express inhibitory factors that can suppress T cell proliferation in a contact independent manner, while it has no effect on the proliferation of the lymphoma cells. One of the inhibitory molecules produced by MDV-lymphoma cells is TGF-beta, which has been shown to inhibit T cell function *in vitro* [19]. To determine whether TGF-beta produced by the tumour cells can also exert inhibitory effects on T cells, splenocytes isolated from RIR birds were stimulated with Con-A in the presence of the tumour supernatant (5% of total volume) and anti-TGF-beta blocking antibody or a control antibody. The results demonstrate that anti-TGF-beta antibody could not restore T cell function, indicating that other factors secreted by those tumour cells are likely to be more important. To examine the role of COX-2/PGE2 pathway in the inhibitory effects of MDV-induced lymphoma cells, splenocytes isolated from RIR birds were stimulated with Con-A in the presence of the tumour supernatant (5% of total volume) with or without cyclooxygenase-2 (COX-2) inhibitor (SC-236) and anti-TGF-beta blocking antibody. The results demonstrate that COX-2 inhibitor can partially restore T cell proliferation and treatment with both COX-2 inhibitor and anti-TGF-beta blocking MAb further increased Con A-induced T cell proliferation (Fig 7I).Taken together, our data demonstrate that PGE2 and TGF-beta are involved in the inhibition of T cell function in a contact independent manner.

## Discussion

Early studies identified a population of human and murine CD4^+^ T cells that co-express high levels of CD25, IL-2 receptor α-chain, and demonstrated that these cells are essential for maintenance of peripheral self-tolerance [20]. Because activation of T cells can lead to upregulation of CD25 molecules, subsequent studies proposed Foxp3, a member of the fork-head/winged-helix family of transcriptional factor, as a unique marker for identification of human and murine naturally occurring Treg cells. However, activated human T cells may also transiently expressed Foxp3 [21, 22]. The presence of Foxp subfamily members including Foxp3 is limited to jawed vertebrates and Foxp3-like genes are not found in chicken [5]. Here we show that a subpopulation of chicken CD4^+^ T cells express membrane bound TGF-beta, an inhibitory cytokine that possess immune-regulatory properties. The results demonstrate that TGF-beta^+^CD4^+^ T cells are distinct from CD4^+^CD25^+^ T cells as the majority of TGF-beta^+^CD4^+^ T cells do not express CD25 molecules.

Intracellular signalling within human and murine Treg cells differs from that of effector T cells. In the latter, activation of AKT results in cytoskeletal rearrangements, cytokine production, cell-cycle progression and engagement of T-cell effector functions. In contrast, Treg cells that are functionally hypo-responsive have a defect in AKT pathway [14], and AKT activation can impair *de novo* induction of Treg cells by TGF-beta [23, 24]. Our data show that chicken TGF-beta^+^ Treg cells have a defect in AKT phosphorylation, confirming that these cells are hypo-responsive. In this study, we also demonstrated that chicken TGF-beta^+^ Treg cells are present in the spleen, blood and lung of naïve birds, but, unexpectedly, these cells were not found in the thymus. This suggests that chicken TGF-beta^+^ Treg cells are induced in the periphery and that thymic Treg cells do not express membrane bound TGF-beta. However, our result does not exclude the possibility that other cells in the thymus, *e*.*g*. apoptotic thymocytes, may express and release TGF-beta which is known to be required for T cell development in the thymus [25, 26]. Induction of TGF-beta^+^ Treg cells in the periphery could be induced by stimulants such as TLR ligands and metabolites produced by gut microbial fermentation [27–29]. Similar to human Treg cells, chicken TGF-beta^+^ Treg could be induced via activation of non-Treg cells with plate-bound anti-CD3 antibody *in vitro*. However, it is unclear how exactly TGF-beta^+^ Treg cells can be induced *in vivo*. It is possible that metabolites such as short chain fatty acids produced by gut microbial fermentation induce TGF-beta^+^ Treg cells [29]. This notion is supported by our results demonstrating that high frequencies of TGF-beta^+^ Treg cells are found in the largest avian gut associated lymphoid tissue, cecal tonsil (CT). Mononuclear cells from CT were hypo-responsive and expressed inhibitory molecules, suggesting that gut associated lymphoid tissues have an immuno-privileged microenvironment in the chickens, and these cells can modulate the inflammatory response. Further work is required to determine the exact mechanism involved in the induction of large numbers of TGF-beta^+^ Treg cell in gut associated lymphoid tissues in chickens and study the role of gut TGF-beta^+^ Treg cells in health and diseases of intestinal tract in chickens. The main function of TGF-beta^+^ CD4^+^ T cells in gut associated lymphoid tissues may be to act as T follicular helper cells and to provide help for B cells to produce IgA, as well as suppress pro-inflammatory responses.

Viruses can trigger and expand both nTreg and induced regulatory T cells (iTregs) via poorly understood mechanisms. It is believed that non-TCR mediated mechanisms, *e*.*g*. innate ligands, may be involved in the induction of Treg cells. For example, it has been suggested that hepatitis C and influenza viruses can increase circulating bioactive TGF-beta levels by activating latent TGF-beta [30, 31], an inhibitory cytokine which can induce Treg cells. On the other hand, some viruses activate Treg cell expansion via ligation of TLR, induction of IL-2 and generation of inhibitory antigen presenting cells [32]. In this study, we demonstrated that RB1B (very virulent MDV) can induce the expansion of Treg cells in the susceptible chicken lines. Further studies are required to examine the effect of mild MDV or very virulent plus MDV on Treg cell activation/expansion. Based on our data, it can be speculated that the formation of tumour lesions in the host is associated with activation/expansion of Treg cells. High mortality rates observed in the chickens infected with very virulent plus MDV may occur prior to the formation of tumour. This may affect the expansion of Treg cells, however further experiments are required to determine the effects of these viruses on Treg cell expansion/activation. Here, we demonstrated that chicken lines that are genetically susceptible to Marek’s Disease (MD) (line P and line 7) have intrinsically higher concentrations of TGF-beta^+^ Treg cells. At the time of these experiments, we had no access to tissues from line 6 (another resistant line with similar MHC molecules as line 7) chickens. Therefore, we were unable to examine the frequencies of intrinsic TGF-beta Treg cells in these birds. However, these experiments are planned and the transfer of TGF-beta Treg cells from line 7 (susceptible:B2 haplotype) to line 6 (resistant:B2 haplotype) chickens will also provide further information on the role of TGF-beta Treg cells in susceptibility to the disease. Our current results demonstrate an association between susceptibility to virus-induced lymphoma and concentrations of Treg cells. This hypothesis is in agreement with reports indicating that Treg cells can confer susceptibility to pathogens and the removal of Treg cells protects the animals from the disease [33–36]. In our study, we did not observe any differences in percentages of CD4^+^CD25^+^ or CD4^+^CD25^high^ T cells in the chicken lines, indicating that only TGF-beta^+^ Treg cells are associated with susceptibility to MDV. But how can TGF-beta^+^ Treg cells contribute to increased susceptibility to MDV? It is possible that TGF-beta^+^ Treg cells dampen down the inflammatory response induced by MDV infection, leading to an increase in virus replication and expression of viral genes involved in the transformation. This notion is supported by the results demonstrating that higher MDV replication occurs in the susceptible chicken lines compared to the resistant lines [11]. Since MDV is a cell-associated virus, it is believed that cell-mediated immunity plays an important role in control of the disease. A suppressive effect of TGF-beta^+^ Treg cells on cell-mediated immunity will result in enhanced susceptibility to the disease. The expression of TGF-beta by MDV-transformed T cells indicates that these cells may exert their immuno-regulatory properties by production of this inhibitory cytokine. TGF-beta inhibits activation and proliferation of naïve T cells, and induces apoptosis in activated T cells [37]. On the other hand, T cells exposed to TGF-beta and IL-6, which is produced during inflammatory response, can develop into Th17 cells, known to be involved in immunopathology [38]. It has been shown that IL-6 gene expression is upregulated in chicken lines that are susceptible to Marek’s Disease. [39]. Therefore, it is possible that induction of TGF-beta and IL-6 in the MDV-infected susceptible birds can lead to the development of Th17 cells. In addition to the immunological effects of TGF-beta, this cytokine can induce apoptosis of various cells including virus-infected cells or transformed cells. How do MDV-induced lymphoma cells escape anti-proliferative effects of autocrine and paracrine TGF-beta? Some cancer cells escape the tumour suppressor effects of TGF-beta by down-regulation/mutation of the type 1 and 2 receptors, Smad2 and Smad4 [40]. It has been shown that a viral analog of cellular miR-155 in MDV, which is critical for oncogenicity of MDV, down-regulate TGF-beta signalling [41]. In agreement with this report, our results show that MDV-transformed TGF-beta^+^ lymphoma cells down-regulate TGF-beta receptors, altering tumour suppressive activity of TGF-beta to tumour-promoting activity. Lymphoma cells may release many inhibitory factors (TGF-beta, VEGF, PGE2, *etc*.) which can cause immunosuppression. Here we demonstrated that COX-2 inhibitor can partially restore the inhibitory effects of soluble factors released by the tumour cells confirming that MDV-induced tumour cells modulate the function of immune cells via COX-2/PGE2 pathway. In addition, we observed that anti-TGF-beta have synergistic effect with COX-2 inhibitors, while has no or very negligible effect when it is used on its own. The results indicate that PGE2 and TGF-beta released by lymphoma cells may be involved in the immunosuppression.

**Fig 8:**
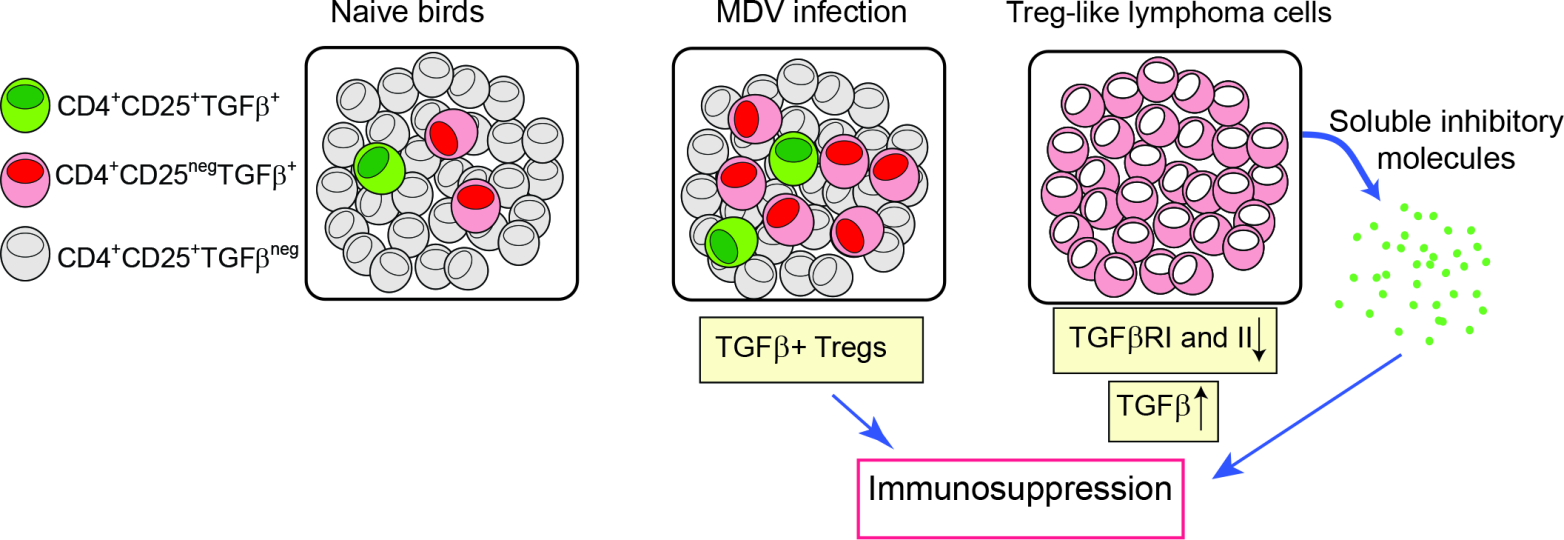
Schematic of immunosuppression via induction of TGF-beta^+^ Treg during MDV infection. Upon infection with MDV via respiratory route, TGF-beta^+^ Treg cells, but not CD4^+^CD25^+^TGF-beta^neg^T cells, are expanded at 4 dpi in the lungs and at day 21 post infection in the spleens. There is an association between the induction of TGF-beta^+^ Treg cells and immunosuppression/pathogenicity of the disease. TGF-beta^+^ MDV-induced lymphoma cells express low levels of TGF-betaRI and II, while produce soluble inhibitory factors which can induce immunosuppression.

One of the prominent features of Marek’s Disease (MD) is a generalized immuno-suppression in chickens. The exact mechanism responsible for MDV-induced immuno-suppression has not yet been defined [11]. As summarized in the proposed model shown in Fig 8, we demonstrate that the induction and expansion of TGF-beta^+^ Treg cells are involved in MD-induced immunosuppression. It should be noted that Treg cells and TGF-beta producing tumour cells are probably induced by different mechanisms and are not necessarily the same in either phenotype or behaviour. The effects of MDV infection on induction/expansion of TGF-beta^+^ Treg cells suggest that these cells may be involved in pathogenesis of the disease, and expansion of these cells can contribute to MDV-induced immunosuppression. This notion is supported by observation that the MDV vaccine strain (CVI988-Rispens) which shares a 98% homology with virulent MDV strains does not trigger the induction of TGF-beta^+^ Treg cells. Furthermore, we demonstrate that MDV-induced tumour cells produce PGE2 and TGF-beta and perhaps other soluble factors that can inhibit T cell function, and may contribute to MDV-induced immunosuppression after transformation phase of the disease. Taken together, our results indicate that Treg cells may be involved in MDV-induced immunosuppression at early stage of infection and soluble factors produced by MDV-induced lymphoma cells can cause immunosuppression.

## Materials and methods

### Ethics statement

All animal experiments were conducted based on the guidelines and care approved (project license PPL 30/3169) by the UK government Home Office and the personnel involved in the procedures had obtained personal license from the UK Home Office.

### Animal experiments

SPF inbred chicken lines including line P (homozygous for the B19 haplotype), line N (homozygous for the B21 haplotype), line 7 (homozygous for the B2 haplotype), and Rhode Island Red (RIR; outbred chicken line) were raised and kept at isolation unit at The Pirbright Institute. Tissues (spleen, lungs, cecal tonsil, and thymus) were harvested after cervical dislocation of the birds. In total, 40 one-day-old line P birds were inoculated with dusts via intra-tracheal route using a Penn-Century device. Twenty a-day old line P birds per group were inoculated with 5 mg of RB1B-containing chicken dust via intra-tracheal route or mock-infected controls. The dust was collected from our previous experiment in which one-day-old RIR birds were infected with CEF cells-RB1B (1,000 PFU) or mock-infected CEF cells via intra-abdominal route. MDV-genome copies were confirmed in the dust using qPCR as previously described [42]. Five MDV-infected birds and 5 mock-infected birds were taken at 4, 11, 21 and 28 dpi. Replication of MDV (RB1B) was confirmed by analyzing genome copy numbers were above 1.00E+3 in spleens of birds using qPCR. MDV-infected birds showed splenomegaly in post-mortem examination on 21 and 28 dpi. Day-old Rhode Island Red chickens were inoculated with 1,000 PFU CEF cells-RB1B (oncogenic virus; n = 8), 1,000 PFU CEF cells-CVI988/Rispens (vaccine strain; n = 8), or mock infected-CEF cells (controls only; n = 8) via the intra-abdominal route, and splenocytes were harvested on 21 dpi for further analysis. Day-old line N chicks (n = 3) were injected with 1,000 PFU RB1B via intra-abdominal route, and the frequencies of TGF-beta^+^ Treg cells in the spleens were analysed 3-weeks post infection.

### Virus preparation

A day-old Rhode Island Red (RIR) chickens were infected via the intra-abdominal route with 1,000 plaque forming units (PFU) of RB1B. Splenocytes were harvested at 14 dpi and were co-cultured with primary CEF cells for 7 days. Once the cytopathic effects were observed, the cell-associated MDV-infected CEF cells were passaged two times in fresh CEF to prepare virus stocks. The virus stocks were titrated and stored in liquid nitrogen. Commercial CVI988/Rispens vaccine virus (Nobilis Rismavac) was obtained from Intervet.

### DNA preparation and real-time PCR

Dust samples were prepared as 5 mg aliquots and total DNA was isolated from dust samples using a DNeasy kit (Qiagen, Manchester, UK). For absolute quantification of MDV genome copies, we performed real-time quantitative duplex PCR (q-PCR) for the detections of the MDV *meq* gene in DNS isolated from dust and splenoyctes. The chicken *ovotransferrin* (*ovo*) was used as a reference gene as previously described [42]. Ten microgram bovine serum albumin was added to reactions using dust samples to block the inhibitory effect of melanin pigment. MDV genomes (for 10^4^ cells or 1 μg dust) were quantified using standard curves which were calibrated against plasmid contracts of known target gene copy number.

### Relative quantification of cytokine expression in cecal tonsils

Total RNA was isolated from CD4^+^ T cells isolated from splenocytes or cecal tonsils after stimulation with PMA (50 ng/ml) and ionomycin (100 ng/ml) at 41°C, 5% CO2 for 4 hrs. The RNA was quantified and treated with DNA-free DNA removal kit (Thermo Fisher Scientific, Paisley, UK) for removal of genomic DNA contamination and reverse transcribed into cDNA with maxima H Minus First Strand cDNA Synthesis Kit according to the manufacturer's instruction (Thermo Fisher Scientific, Paisley, UK). Primer sequences for all target cytokines and housekeeping genes are enlisted in S1 Table. The SYBR green based real-time PCR was performed with a LightCycler 480 SYBR Green I Master kit (Roche Applied Science, USA). The Quantitative PCR was performed in QuantStudio5 (Thermo Fisher Scientific, Paisley, UK) with the following conditions: initial denaturation was performed at 95°C for 1 min, followed by 40 cycles of denaturation at 95°C for 15s and annealing / extension at 60°C for 30s, with end point melt-curve analysis. The relative fold change of target genes in cecal tonsils was calculated by 2−ΔΔCT method. The Ct value for each sample was normalized against GAPDH housekeeping gene for respective sample. The amplified TGF-beta receptor I and II were cloned in pGEMT-easy vector (Promega, USA) and used for generation of the standard curve equation to calculate the absolute copy number of the genes.

### Flow cytometry/ Phosflow

Mononuclear cells isolated from spleens, lungs; cecal tonsils, blood, and thymus were stained with fluorochrome-conjugated monoclonal antibodies, and the expression of surface molecules were analysed using flow cytometry. Prior to staining, the cells were incubated with chicken serum to block FC receptors, and then the cells were incubated with anti-CD4-PE (Cambridge Bioscience, Cambridge, UK), anti-TGF-beta1,2,3 (R&D systems, Abingdon, UK), anti-CD25 FITC mAbs (Bio-Rad, Watford, UK) at 4°C for 15 min. The cells were washed twice and 7-AAD staining was used to exclude dead cells. The minimal number of 100,000 cells was acquired for FACS analysis. Cells were acquired on a MACSQuant analyser and the data were processed by FlowJo V10 software.

For Phosflow analysis of the cells, mononuclear cells were plated at 5.0 x10^5^ cells per well and stimulated (15 min; 41°C, 5% CO2) with PMA (50 ng/ml) or cell culture media only. The staining was conducted according to the manufacturer’s instruction as previously described [43]. After fixation of the cells for 30 minutes using the fixation/ permeabilization kit (Thermo Fisher Scientific, Paisley, UK), the cells were stained with anti-TGF-beta (R&D Systems, Abingdon, UK) and anti-CD4 mAbs (Cambridge Bioscience, Cambridge, UK), and were permeabilized using Perm buffer for 10 min at 4°C, followed by staining with anti-AKT (S473) PE-conjugated mAbs (BD Bioscience, Oxford, UK) at 4°C for 30 minutes. The cells were washed twice in Perm buffer and re-suspended in FACS buffer and acquired using flow cytometry.

### Inhibitory T cell function assay

The inhibitory effects of TGF-beta^+^ Treg cells on T cell proliferation were analysed in an *in vitro* inhibition assay. TGF-beta^+^ Treg and TGF-beta^-^ CD4^+^ T cells were purified from splenocytes using anti-PE and anti-APC micro-bead kits according to the manufacturer’s instructions (Miltenyi Biotec, Woking, UK). TGF-beta^+^ Treg cells or control cells were co-cultured with the responder cells (CFSE-labelled splenocytes [depleted from TGF-beta^+^ Treg cells]; 3 X 10^4^) at an effector/responder cell ratio of 1:1 in three replicates. The cells were stimulated with 2.5μg/ml Con-A to induce T cell proliferation. In each experiment, the cells from 7 birds were pooled to isolate enough cells for the inhibition assays. T cell proliferation was analysed using flow cytometry at 72 hrs after the co-culture, and proliferation index was calculated using FlowJo v10.07.

For assessing the inhibitory effects of *in vitro* generated TGF-beta^+^ Treg cells, the responder cells (3 x 10^4^ cells/ well) were co-cultured with TGF-beta^+^ Treg cells or TGF-beta^-^ CD4^+^ T cells at an effector/responder ratio of 1:1 in three replicates as described above. The numbers of live cells (7AAD negative) in the culture were counted using flow cytometry.

To study the role of PGE2 and TGF-beta in the inhibitory effects, the cells were treated with 10 μM SC-236 (COX-2 inhibitor) (Tocris, Abingdon, UK) and /or 1 μg/ml anti-TGF-beta blocking antibody (clone 1D11) or a control MAb (mouse IgG1) (R&D Systems, Abingdon, UK) prior to Con-A stimulation (2.5μg/ml).

### Immunofluorescence microscopy

MDV-derived lymphoma cell lines were adhered to coverslips using Cell-Tak adhesive (BD Bioscience, Oxford, UK). Tissues were immediately frozen in isopentane mixed with dry ice, and the frozen samples were kept at -80°C. Tissue sections were cut at 8μm and mounted on gelatin-coated histological slides. Sections were fixed 4% paraformaldehyde at Room temperature for 1 hr and permeabilized in 0.1% Triton X100 in PBS for 10 minutes. After rehydration and blocked (0.5% BSA in PBS), the tissue sections or cells were incubated with mouse anti-chicken CD4 (IgG2b) (Cambridge Bioscience, Cambridge, UK), and mouse anti-TGF-beta (IgG1) (R& D Systems, Abingdon, UK) overnight at 2–8°C. An isotype matched control (R&D Systems, Abingdon, UK) was used to identify non-specific staining. After three washes in PBS at room temperature, the tissues/ cells were incubated with fluorochrome-labeled anti-mouse IgG1 or IgG2b antibodies (Thermo Fisher Scientific, Paisley, UK), and DAPI solution was added for 10 minutes at room temperature. The sections were mounted onto glass slides using Vectashield mounting medium, and coverslips were sealed with nail varnish before visualisation by confocal microscopy (Leica Microsystems).

### Chicken IFN-gamma ELISPOT assay

Chicken ELISPOT assay was performed as described previously [43]. Briefly, nitrocellulose-backed plates were coated with anti-chicken IFN-gamma capture antibody overnight at 4°C. The wells were washed and blocked using RPMI1640 containing 2% FCS (1hr at 41°C). Mononuclear cells were seeded (3 x10^6^ cells/ml) and stimulated with Phorbol Myristate Acetate (PMA; 50 ng/ml) and ionomycin (100 ng/ml) in six replicates and incubated at 41°C for 18hrs. The wells were washed with PBS and distilled water, and then incubated with anti-chicken IFN-gamma biotin (1 μg / ml) (Thermo Fisher Scientific, Paisley, UK) for 1 h. After several washes, the plate was incubated with 100 μl of streptavidin-HRP (1:1000) at 41°C for 1 h. The assay was developed with AEC substrate as per manufacturer’s instruction (BD Bioscience, Oxford, UK). The reaction was terminated by washing plates under running water. The spots were enumerated in the dried plate with ELISPOT reader (Advanced imaging devices, GmbH, Germany). The counted spots were finally expressed as spot forming units (SFU) per million mononuclear cells.

### Cell culture and MDV-derived T cell lines

MDV-transformed CD4^+^ T-cell lines were cultured (41°C, 5% CO2 incubator) in RPMI 1640 growth medium (Thermo Fisher Scientific, Paisley, UK); supplemented 100 μg/mL of penicillin/streptomycin, 10% fetal calf serum, 10% tryptose phosphate broth, 1 mM sodium pyruvate; (Sigma-Aldrich, Dorset, UK), and 50 μM 2-mercaptoethanol (Thermo Fisher Scientific, Paisley, UK).

### Statistical analyses

Statistical calculation included Wilcoxon and Mann Whitney non-parametric to determine significance. Results were considered statistically significant at *P* < 0.05 (*).Statistical analysis of the data was performed using Graph Pad Prism 7. The data are presented as mean +-SD.

## Supporting information

S1 Fig (A) Representative of SSC-FSC and gating strategy of mononuclear cells isolated from spleen, peripheral blood, cecal tonsil, and lung are shown. (B) Microscopy of mononuclear cells isolated from cecal tonsil is demonstrated.
(TIF)

S2 Fig (A) CFSE histograms from T cell proliferation of splenocytes in response to different concentrations of Con-A or PMA are shown. (B) Graphical representation of proliferation index in response to Con-A or PMA.
(TIF)

S1 Table List of primers used for real-time PCR. (DOCX)
