# Supplementary figures and images for: Association of Marek’s Disease induced immunosuppression with activation of a novel regulatory T cells in chickens
Source: PLoS Pathog. 2017 Dec 21;13(12):e1006745. doi: 10.1371/journal.ppat.1006745 (PMC5739506; doi:10.1371/journal.ppat.1006745)

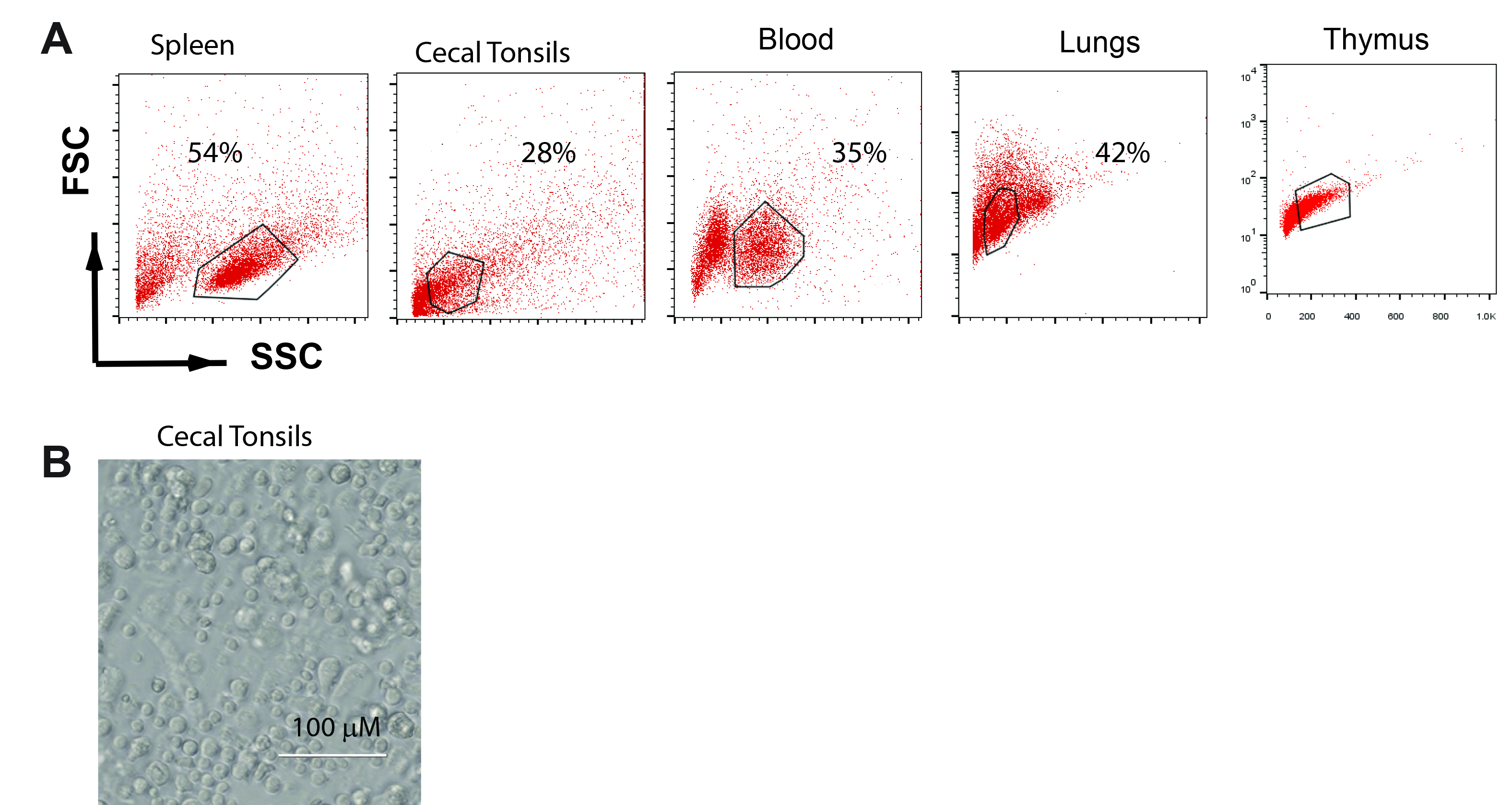

Supplement: S1 Fig — (A) Representative of SSC-FSC and gating strategy of mononuclear cells isolated from spleen, peripheral blood, cecal tonsil, and lung are shown. (B) Microscopy of mononuclear cells isolated from cecal tonsil is demonstrated. (TIF) [file ppat.1006745.s001.tif]

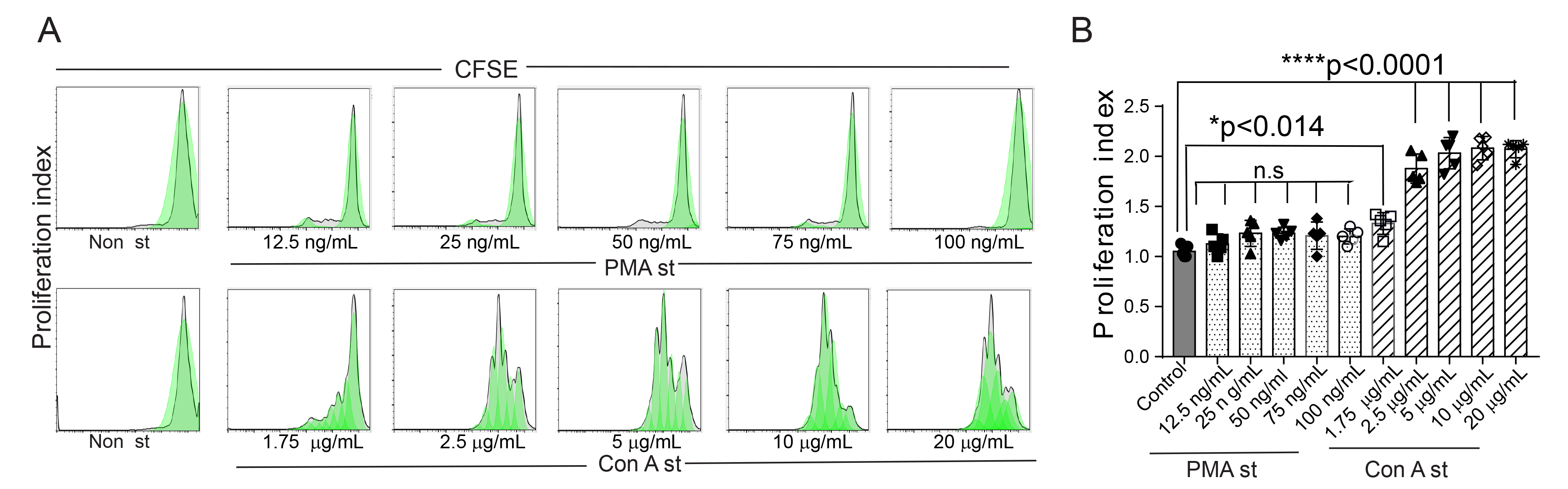

Supplement: S2 Fig — (A) CFSE histograms from T cell proliferation of splenocytes in response to different concentrations of Con-A or PMA are shown. (B) Graphical representation of proliferation index in response to Con-A or PMA. (TIF) [file ppat.1006745.s002.tif]
