# Supplementary material for: Association of Marek’s Disease induced immunosuppression with activation of a novel regulatory T cells in chickens
Source: PLoS Pathog. 2017 Dec 21;13(12):e1006745. doi: 10.1371/journal.ppat.1006745 (PMC5739506; doi:10.1371/journal.ppat.1006745)
Supplement: S1 Table — (DOCX) [file ppat.1006745.s003.docx]

| **Primer name** | **Forward Sequence (5’-3’)** | **Reverse Sequence (5’-3’)** |
| --- | --- | --- |
| **GAPDH** | GGCAGATGCAGGTGCTGAGTAT | CGTCTTCTGTGTGGCTGTGATG |
| **IFN-γ** | AGCTGACGGTGGACCTATTATT | GGCTTT GCGCTGGATTC |
| **CTLA-4** | CAAGATGGAGCGGATGTACC | TGGCTGAGATGATGATGCTG |
| **PD-1** | GTGATTGTGCTGCTGCTCTTTG | GAACTCCAGCACACCGTAGTC |
| **PDL-1** | CTTCACATTACCAGCGTCAGG | GACTGGCATATAAGAGCAAAC |
| **IL-10** | GCTGAGGGTGAAGTTTGAGGAA | TGCTGATGACTGGTGCTGGT |
| **IL-2** | ATCTTTGGCTGTATTTCGGTAG | TGGGTCTCAGTTGGTGTGTAG |
| **TGFBR1** | ggttggcagttaggcatgat | aacatcgacgagcaatttcc |
| **TGFBR2** | aggctggacccttctctctc | gggatgctctcgcactttag |

**sTable 1: List of primers used for real-time PCR.**
